# Higher prevalence of colon polyps in patients with Barrett’s esophagus: a case-control study

**DOI:** 10.1093/gastro/gou050

**Published:** 2014-07-31

**Authors:** Arthi Kumaravel, Prashanthi N. Thota, Hyun-Ju Lee, Tushar Gohel, Mehulkumar K. Kanadiya, Rocio Lopez, Madhusudhan R. Sanaka

**Affiliations:** ^1^Center of Excellence for Barrett’s Esophagus, Department of Gastroenterology and Hepatology, Cleveland Clinic, Cleveland, Ohio, USA and ^2^Department of Biostatistics, Cleveland Clinic, Cleveland, Ohio, USA

**Keywords:** Barrett’s esophagus, colon polyps, cancer prevention

## Abstract

**Background and aims:** Barrett’s esophagus (BE) and colorectal neoplasms share similar risk factors. Previous studies have shown variable prevalence of colon polyps in patients with BE. Our aims were to determine the prevalence and incidence of colon polyps in patients with BE, compared to those without BE.

**Methods:** In this case-control study, the study group included patients, aged 50–75 years, with biopsy-proven BE, who underwent colonoscopy at Cleveland Clinic from January 2002 to December 2011. The control group consisted of age- and sex-matched patients who underwent colonoscopy and also an endoscopy with no evidence of BE during the same time period. Exclusion criteria for both groups were family- or personal previous history of colon cancer or polyps, prior colonic resection, inflammatory bowel disease and familial polyposis syndromes. Patient demographics, comorbidities, medication use and endoscopic and colonoscopic details were collected, including biopsy results.

**Results:** A total of 519 patients were included in the study; 173 patients with BE in the study group and 346 without BE in the control group. Mean age at index colonoscopy was 61 ± 8 years and 75% of patients were male. On index colonoscopy, patients with BE were more likely to have polyps than controls (45% *vs* 32%, respectively; *P = *0.003). Patients underwent between one and five colonoscopies during the follow-up. On multivariate analysis—after adjusting for age, gender and diabetes—patients with BE were 80% more likely to have any type of polyp, and 50% more likely to have adenomas found during colonoscopy.

**Conclusions:** Patients with BE had higher prevalence and incidence of colon polyps. This has important clinical implications for screening and surveillance in BE patients.

## INTRODUCTION

Barrett’s esophagus (BE) is a pre-malignant condition, characterized by replacement of the normal squamous epithelium of the esophagus by columnar epithelium with specialized intestinal metaplasia. Due to increased risk of esophageal adenocarcinoma (EAC) in patients with BE, routine surveillance is recommended. Besides EAC, BE is also reported to be associated with an increased risk of colon polyps and colorectal cancer (CRC). Sontag *et al.* first proposed that there was an association between BE and CRC [1]. Since that proposal, several studies have reported an association between BE and colorectal neoplasia [2–8], while others have found no such association [9–16]. Some of the studies reported that EAC carried a higher risk of colonic neoplasia than esophageal squamous cancer [4, 6]. Even large, population-based studies have shown conflicting findings, with some indicating increased risk of colonic neoplasia [3, 6–8], while others did not [14, 15]. The difficulty in interpreting those studies is due to the small number of patients in some studies [1, 2, 9–13] and lack of true control groups in others [1, 12, 13]. There is also a possibility of bias, since patients with BE were in a surveillance program and therefore more likely to have colonoscopies.

Since both CRC and EAC are rare occurrences, it may be more prudent to evaluate for any association between their more prevalent precursor conditions, i.e. colon polyps and BE. Our aims were therefore to determine (i) whether there is increased prevalence of colon polyps in patients with BE and (ii) whether there is an increased incidence of colon polyps in patients with BE whilst in a surveillance program.

## METHODS

This was a case-control study, performed at the Cleveland Clinic between January 1, 2002 and December 31, 2011. The study group consisted of patients in the BE registry aged 50–75 years, who underwent a colonoscopy at the Cleveland Clinic. The control group was derived from patients aged 50–75 years who had, during the same study period, undergone both an esophagogastroduodenoscopy (EGD) that showed no evidence of BE and a colonoscopy. Groups were frequency matched (1:2), based on gender and age at first colonoscopy. Patients were excluded from either group if they were in a high-risk group (if they had a family history of colon cancer or colon polyps, history of inflammatory bowel disease (IBD), familial polyposis syndromes or prior history of colon polyps), or had a history of colectomy prior to the study period, incomplete colonoscopy or inadequate bowel preparation at colonoscopy. This study was approved by Cleveland Clinic Institutional Review Board.

Each patient’s age, sex, body mass index (BMI), medication use, smoking history, alcohol history and comorbidities were recorded. BE was defined as the presence of columnar-appearing epithelium of any length in the esophagus on endoscopy with specialized intestinal metaplasia on biopsy. Endoscopic features were noted, such as length of BE, size of the hiatal hernia and histological findings. Also, from the colonoscopy reports, information was collected relating to the quality of bowel preparation, completeness of the procedure, number of polyps detected, size, location and histology of each polyp. Polyps were classified into hyperplastic and non-hyperplastic polyps, which included adenomas and sessile serrated adenomas. Adenomas included tubular adenomas, tubulovillous adenomas and adenomas with high-grade dysplasia. For statistical analysis, the proximal colon included the cecum, the ascending- and transverse colon, including the splenic flexure. The colon distal to this was defined as the distal colon.

### Statistical analysis

Data were presented as mean ± standard deviation, median (25^th^ and 75^th^ percentiles) or *n* (%). For each patient, index colonoscopy findings were reported, as well as lifetime overall colonoscopy findings (based on all colonoscopies reported). A univariate analysis was performed to assess differences between subjects with and without BE. Analysis of variance (ANOVA) or the non-parametric Kruskal-Wallis tests were used for continuous or ordinal factors and Fisher’s exact test or Pearson’s chi-squared test were used to compare categorical variables. In addition, generalized linear models (GLM) with a logit link for binary outcomes were used to model the presence of polyps in any colonoscopy performed, while accounting for multiple procedures per patient. An autoregressive (AR1) covariance structure was used to model the intra-subject correlation. The presence of any polyp was modeled as the outcome with BE, age, gender and diabetes as the independent predictors. The same was done for presence of non-hyperplastic polyps, hyperplastic polyps, adenomas and SSA. A *P*-value <0.05 was considered statistically significant. All analyses were performed using SAS (version 9.2; The SAS Institute, Cary, NC, USA).

## RESULTS

### Patient population

A total of 519 patients were included in the study. The cases were 173 patients with BE who underwent colonoscopy during the study period. The control group were age- and sex-matched controls with endoscopic confirmation of the absence of BE, and comprised 346 patients. Seventy-five percent of the patients were male and mean age at the time of first colonoscopy was 61 ± 8 years. The most common indication for colonoscopy was average risk screening (55.2%), followed by gastrointestinal bleeding (12.3%), abdominal pain (8.4%), diarrhea (7.8%), constipation (3.5%) and other (12.6%). The most common indication for EGD in control group was abdominal pain (23%), followed by gastroesophageal reflux disease (21%), gastrointestinal bleeding (12%), dyspepsia (9%), nausea (4%) and weight loss (4%). Mean length of BE was 3.3 ± 2.6 cm. Mean hiatal hernia size was 3.2 ± 1.6 cm. Patients with BE were more likely to be caucasian, to use proton pump inhibitors (PPI), and less likely to have *diabetes mellitus* than controls. There were no significant differences between the two groups in terms of age, BMI, smoking history, alcohol use, aspirin/non-steroidal anti-inflammatory drugs (NSAID) or statin use and comorbidities such as hypertension model hyperlipidemia (Table 1). Among the BE cohort, 147 patients had non-dysplastic BE, 17 had indefinite indications for dysplasia or low-grade dysplasia, 8 had high-grade dysplasia and one patient had intramucosal cancer in the BE segment.

**Table 1.** Demographic and clinical characteristics

Factors	*n* missing	No Barrett’s esophagus (*n = *346)	Barrett’s esophagus (*n = *173)	*P*-value
Male (*n*, %)	3	256 (74.6)	129 (74.6)	0.99
Caucasian (*n*, %)	15	266 (78.9)	152 (91.0)	***<0.001***
Age at index EGD (*n*, %)	2	60.9 ± 8.3	59.9 ± 8.3	0.2
Age at index colonoscopy (years)	5	60.6 ± 8.3	60.5 ± 7.8	0.95
BMI (kg/m^2^)	19	28.7 ± 6.3	29.1 ± 6.5	0.53
Smoking (*n*, %)	17			0.3
Non smoker		146 (43.7)	66 (39.3)	
Ex-smoker		158 (47.3)	91 (54.2)	
Current smoker		30 (9.0)	11 (6.5)	
Alcohol use (*n*, %)	36			0.57
Never		127 (39.7)	59 (36.2)	
Mild (<7 drinks/week)		152 (47.5)	83 (50.9)	
Moderate (7–14 drinks/week)		19 (5.9)	14 (8.6)	
Severe (>14 drinks/week)		7 (2.2)	2 (1.2)	
Ex-alcohol user		15 (4.7)	5 (3.1)	
Medications (non-exclusive) (*n*, %)				
Aspirin		151 (43.6)	79 (45.7)	0.66
NSAID		45 (13.0)	22 (12.7)	0.93
Statins		164 (47.4)	81 (46.8)	0.9
PPI		171 (49.4)	150 (86.7)	***<0.001***
H2 blockers		18 (5.2)	8 (4.6)	0.78
Comorbidities (non-exclusive) (*n*, %)				
Hypertension		175 (50.6)	103 (59.5)	0.054
Diabetes		80 (23.1)	27 (15.6)	***0.046***
Hyperlipidemia		172 (49.7)	82 (47.4)	0.62

BMI = body mass index; EGD = esophagogastroduodenoscopy; NSAID = nonsteroidal anti-inflammatory drugs; PPI = proton pump inhibitors. *P*-values < 0.05 are shown in italics.

### Findings on index colonoscopy

On index colonoscopy (the first colonoscopy during study period), patients with BE were more likely than the controls to have polyps (45.1% *vs* 31.8%, respectively; *P = *0.003). Hyperplastic polyps were more common in patients with BE (21% *vs* 9%, respectively; *P < *0.001). Also, there was increased prevalence of both proximal and distal polyps in patients with BE. There were no significant differences in the prevalence of adenomas between study and control groups (Table 2). One patient in the control group was found to have colon cancer on index colonoscopy. Dysplasia in BE did not confer any higher risk of colon polyps than non-dysplastic BE (Table 3).

**Table 2.** Index colonoscopy findings

Factors	No Barrett’s esophagus (*n = *346)	Barrett’s esophagus (*n = *173)	*P*-value
Any polyps (*n*, %)	110 (31.8)	78 (45.1)	***0.003***
Non-hyperplastic polyps (*n*, %)	86 (24.9)	53 (30.6)	0.16
Hyperplastic polyps (*n*, %)	31 (9.0)	36 (20.8)	***<0.001***
Any adenomas (*n*, %)	85 (24.6)	53 (30.6)	0.14
Sessile serrated adenomas (*n*, %)	3 (0.9)	4 (2.3)	0.23
Colorectal cancers (*n*, %)	1 (0.3)	0 (0.0)	0.99
Distal polyps (*n*, %)	60 (17.3)	49 (28.3)	***0.004***
Proximal polyps (*n*, %)	67 (19.4)	47 (27.2)	***0.043***

*P*-values less than 0.05 are shown in italics.

**Table 3.** Index colonoscopy findings based on dysplasia in Barrett’s esophagus

Factors	No Barrett’s esophagus (*n = *346)	Barrett’s esophagus without dysplasia (*n = *129)	Barrett’s esophagus with dysplasia (*n = *44)	*P*-value
Any polyps (*n*, %)	110 (31.8)	60 (46.5)**^a^**	18 (40.9)	***0.01***
Non-hyperplastic polyps (*n*, %)	86 (24.9)	41 (31.8)	12 (27.3)	0.32
Hyperplastic polyps (*n*, %)	31 (9.0)	30 (23.3)**^a^**	6 (13.6)	***<0.001***
Any adenomas (*n*, %)	85 (24.6)	41 (31.8)	12 (27.3)	0.28
Sessile serrated adenomas (*n*, %)	3 (0.9)	4 (3.1)	0 (0.0)	0.18F
Colorectal cancers (*n*, %)	1 (0.3)	0 (0.0)	0 (0.0)	0.99F
Distal polyps (*n*, %)	60 (17.3)	41 (31.8)**^a^**	8 (18.2)	***0.002***
Proximal polyps (*n*, %)	67 (19.4)	33 (25.6)	14 (31.8)	0.089

^a^Significantly different from No Barrett’s esophagus.

A significance level of 0.017 was used for pairwise *ad hoc* comparisons.

### Findings on follow-up colonoscopies

Patients underwent one to five colonoscopies during the follow-up period. Subjects with BE were more likely than the controls to have two or more colonoscopies during follow-up (44% *vs* 21%, respectively; *P < *0.001). Patients with BE had more polyps than controls, including hyperplastic polyps, adenomas and sessile serrated adenomas (SSA). The numbers of follow-up colonoscopies and findings are shown in Table 4. There were no significant differences in the incidence of colon polyps between BE patients with and without dysplasia (Table 5).

**Table 4.** Colon polyps on follow-up colonoscopies

Factors	No Barrett’s esophagus (*n = *346)	Barrett’s esophagus (*n = *173)	*P*-value
No. of colonoscopies (*n*, %)			***<0.001***
1	272 (78.6)	97 (56.1)	
2	63 (18.2)	50 (28.9)	
≥3	11 (3.2)	26 (15.0)	
Any polyps (*n*, %)	118 (34.1)	93 (53.8)	***<0.001***
Non-hyperplastic polyps (*n*, %)	42 (12.1)	50 (28.9)	***<0.001***
Hyperplastic polyps (*n*, %)	92 (26.6)	69 (39.9)	***0.002***
Any adenomas (*n*, %)	91 (26.3)	69 (39.9)	***0.002***
Sessile serrated adenomas (*n*, %)	4 (1.2)	7 (4.0)	***0.031***
Colorectal cancers (*n*, %)	1 (0.3)	0 (0.0)	0.99
Distal polyps (*n*, %)	72 (20.8)	61 (35.3)	***<0.001***
Proximal polyps (*n*, %)	77 (22.3)	66 (38.2)	***<0.001***

**Table 5.** Colon polyps on follow-up colonoscopies based on dysplasia in Barrett’s esophagus

Factors	No Barrett’s esophagus (*n = *346)	Barrett’s esophagus without dysplasia (*n = *129)	Barrett’s esophagus with dysplasia (*n = *44)	*P*-value
No. of colonoscopies (*n*, %)				***<0.001***
1	272 (78.6)	71 (55.0)**^a^**	26 (59.1)**^a^**	
2	63 (18.2)	36 (27.9)	14 (31.8)	
≥3	11 (3.2)	22 (17.1)	4 (9.1)	
Any polyps (*n*, %)	118 (34.1)	72 (55.8)**^a^**	21 (47.7)	***<0.001***
Non-hyperplastic polyps (*n*, %)	92 (26.6)	51 (39.5)**^a^**	18 (40.9)	***0.008***
Hyperplastic polyps (*n*, %)	42 (12.1)	43 (33.3)**^a^**	7 (15.9)	***<0.001***
Any adenomas (*n*, %)	91 (26.3)	51 (39.5)**^a^**	18 (40.9)	***0.007***
Sessile serrated adenomas (*n*, %)	4 (1.2)	5 (3.9)	2 (4.5)	0.095
Colorectal cancers (*n*, %)	1 (0.3)	0 (0.0)	0 (0.0)	0.99F
Distal polyps (*n*, %)	72 (20.8)	50 (38.8)**^a^**	11 (25.0)	***<0.001***
Proximal polyps (*n*, %)	77 (22.3)	48 (37.2)**^a^**	18 (40.9)**^a^**	***<0.001***
No. of polyps (*n*, %)				***<0.001***
0	228 (65.9)	57 (44.2)**^a^**	23 (52.3)	
1	63 (18.2)	22 (17.1)	8 (18.2)	
2	24 (6.9)	18 (14.0)	5 (11.4)	
≥3	31 (9.0)	32 (24.8)	8 (18.2)	
No. of HP (*n*, %)				***<0.001***
0	304 (87.9)	86 (66.7)**^a^**	37 (84.1)	
1	29 (8.4)	24 (18.6)	4 (9.1)	
2	7 (2.0)	10 (7.8)	2 (4.5)	
≥3	6 (1.7)	9 (7.0)	1 (2.3)	
No. of adenomas (*n*, %)				***0.004***
0	255 (73.7)	78 (60.5)**^a^**	26 (59.1)	
1	53 (15.3)	26 (20.2)	8 (18.2)	
2	18 (5.2)	11 (8.5)	6 (13.6)	
≥3	20 (5.8)	14 (10.9)	4 (9.1)	
No. of sessile serrated adenomas (*n*, %)				0.094
0	342 (98.8)	124 (96.1)	42 (95.5)	
1	3 (0.9)	3 (2.3)	1 (2.3)	
2	1 (0.3)	2 (1.6)	1 (2.3)	

^a^Significantly different from No Barrett’s esophagus

A significance level of 0.017 was used for pairwise *ad hoc* comparisons.

Multivariate analysis was performed to identify the effect of BE on the development of colon polyps, after accounting for multiple procedures in the same subject using an autoregressive (AR1) covariance structure and adjusting for age, gender and presence of diabetes. Patients with BE were 80% more likely to have any type of polyp, and 50% more likely to have adenomas (Table 6).

**Table 6.** Effect of Barrett’s esophagus on colonoscopy findings: multivariate generalized linear models^a^

Outcomes	Adjusted^b^ OR (95% CI)	*P*-value
Any polyps	1.8 (1.3–2.6)	***<0.001***
Non-hyperplastic polyps	1.4 (1.0–2.1)	0.053
Hyperplastic polyps	2.6 (1.7–4.1)	***<0.001***
Any adenomas	1.5 (1.0–2.1)	***0.044***
Sessile serrated adenomas	2.6 (0.7–9.2)	0.15
Colorectal cancers	1.6 (1.1–2.3)	***0.017***
Distal polyps	1.9 (1.3–2.8)	***<0.001***

^a^An autoregressive (AR1) covariance structure was used to account for correlation between procedures perfomed on the same subject.

^b^Adjusted for gender, age and diabetes

## DISCUSSION

Our study showed that patients with BE are at greater risk than controls of developing colonic polyps. The relative risk for any type of colon polyps is 1.8 and for adenomas is 1.5. These findings parallel the recent study findings by Sonneberg *et al.* [8]. In that study, from a large histopathological database of 203 534 patients, of which 12 221 had BE, patients with BE had higher prevalence of hyperplastic polyps (OR 2.14; 95% CI 2.02–2.27), adenomatous polyps (OR 2.52; 95% CI 2.41–2.64), and CRC (OR 1.75; 95% CI 1.39–2.22). Irrespective of degree of dysplasia, the association between BE and colon polyps applied similarly to polyps of differing sizes, number and locations within the large bowel. However, they did not have any clinical information on the patients, in terms of family history of colon cancer and other risk factors that might affect the association, and no long term follow-up data. Similar findings have been reported in prior studies looking at the association between BE and colon polyps [1, 2].

There are a few studies suggesting that BE is not associated with increased risk of colorectal neoplasms. In a case-control study of 104 patients with BE and 537 controls, adenomas were found in 26 Barrett’s patients (25%) and 75 controls (14%) [16]. The prevalence of adenomas was greater in the BE group than in the control group (*P* < 0.01) but the relationship became non-significant after adjustment for age and sex by a logistic regression model (OR 1.4; 95% CI 0.7–2.7). In another case-control study of 72 consecutive patients with BE and 27 controls, colorectal adenomas were seen in 17 patients (24%) with BE and in 8 controls (30%). Using a logistic regression model with the occurrence of colonic adenoma as dependent and sex, age and occurrence of BE as explanatory variables, none of these was found to be a significant risk factor for the appearance of colonic adenoma [10]. This may be a type II error, as a recent meta-analysis of seven studies, including the aforementioned and totalling 361 BE cases, showed an increased risk of adenomas in patients with BE, with pooled OR of 1.69 (95% CI 1.20–2.39) [17].

There have been several potential explanations for the association between BE and colon polyps or CRC but the underlying mechanisms responsible for the higher prevalence of colon polyps in BE patients are not clearly understood. Patients with BE are more likely to have some of the same ‘environmental’ risk factors associated with CRC development, such as age, gender, obesity, alcohol consumption and smoking [18]. The genetic pathways leading to colon cancer have been well elucidated; however, the genetic alterations associated with development of BE and its progression to EAC are not as well defined. Certain mutations in the colon adenoma-to-carcinoma pathway, such as mutations to the APC gene and activation of the Src gene, have also been described in BE [19–21]. Both of these factors can activate the COX-2 and increased expression of COX-2 plays a pivotal role in the pathophysiology of EAC and CRC [22]. Other genetic aberrations associated with cancer progression described in both conditions include p53 mutations, as well as allelic loss of chromosomes 17p and 18q [23, 24]. However, these genetic abnormalities have been reported in other cancers, too.

The strength of our study is the large cohort of patients with BE who underwent colonoscopy and had a long follow-up period. Previous studies did not follow patients over time and reported prevalence rates based on one-time colonoscopy results, or lacked a control group. Our control group included only patients who had an EGD that confirmed the absence of BE. In addition, our study and control groups are well matched in terms of confounding variables associated with colon polyps, such as BMI, smoking, alcohol use, aspirin, NSAID and statin use, thus ruling out any diagnostic bias.

The main limitation of this study is that it is a single-center study from a tertiary referral center and, hence, results may not apply to the general population. There is also a small possibility of missing information about a few follow-up colonoscopies that were performed outside of our institution, but every effort had been made to have as complete data as possible. More patients with BE than controls had multiple follow-up colonoscopies, which may raise a question of diagnostic bias. However, this might be because of increased prevalence of colon polyps in BE patients, necessitating more surveillance colonoscopies. Another limitation is the absence of data on waist–hip ratio, which is reflective of central obesity. However, this may not be a confounding factor as there is no difference in BMI between the two groups.

In conclusion, our study confirms the association between BE and colon polyps. BE patients have higher incidence of adenomas while under surveillance, than controls without BE. This association might have important implications for screening and surveillance in these patients. Further studies are needed to determine the appropriate screening and surveillance colonoscopy intervals for patients with BE.

**Conflict of interest:** none declared.
